# Insight Into the Mechanisms and the Challenges on Stem Cell-Based Therapies for Cerebral Ischemic Stroke

**DOI:** 10.3389/fncel.2021.637210

**Published:** 2021-02-25

**Authors:** Huiyong Liu, Sydney Reiter, Xiangyue Zhou, Hanmin Chen, Yibo Ou, Cameron Lenahan, Yue He

**Affiliations:** ^1^Department of Neurosurgery, Tongji Hospital, Tongji Medical College, Huazhong University of Science and Technology, Wuhan, China; ^2^Department of Kinesiology, University of Texas at Austin, Austin, TX, United States; ^3^Department of Biomedical Sciences, Burrell College of Osteopathic Medicine, Las Cruces, NM, United States

**Keywords:** stem cell, cell transplantation, ischemic stroke, brain regeneration, molecular mechanism

## Abstract

Strokes are the most common types of cerebrovascular disease and remain a major cause of death and disability worldwide. Cerebral ischemic stroke is caused by a reduction in blood flow to the brain. In this disease, two major zones of injury are identified: the lesion core, in which cells rapidly progress toward death, and the ischemic penumbra (surrounding the lesion core), which is defined as hypoperfusion tissue where cells may remain viable and can be repaired. Two methods that are approved by the Food and Drug Administration (FDA) include intravenous thrombolytic therapy and endovascular thrombectomy, however, the narrow therapeutic window poses a limitation, and therefore a low percentage of stroke patients actually receive these treatments. Developments in stem cell therapy have introduced renewed hope to patients with ischemic stroke due to its potential effect for reversing the neurological sequelae. Over the last few decades, animal tests and clinical trials have been used to treat ischemic stroke experimentally with various types of stem cells. However, several technical and ethical challenges must be overcome before stem cells can become a choice for the treatment of stroke. In this review, we summarize the mechanisms, processes, and challenges of using stem cells in stroke treatment. We also discuss new developing trends in this field.

## Introduction

Stroke is a leading cause of functional impairment and death worldwide (Meschia et al., 2014). Approximately 795,000 people suffer from a stroke and more than 140,000 people die from it in the United States annually (Virani et al., 2020). There are three types of stroke: transient ischemic attack, ischemic stroke, and hemorrhagic stroke. Approximately 80% of strokes are ischemic (Thrift et al., 2001). Despite the high incidence of stroke, an effective therapy does not exist, particularly for chronic stroke. Currently, treatment for acute ischemic stroke is limited to efficient and fast removal of thrombus *via* intravenous use of tissue-type plasminogen activator (tPA) within 4.5 h, or endovascular mechanical thrombectomy within 6 h after symptom onset (Powers et al., 2018). However, the narrow effective therapeutic window is the major limitation, and there is a high potential for hemorrhagic transformation. Consequently, few patients receive these therapies, which are compounded by the lack of existing rehabilitative therapy. Therefore, there is substantial interest in alleviating the post-stroke sequelae and improving restorative recovery.

Recent developments in stem cell biology have provided renewed hope for treating ischemic stroke. Stem cells are characterized by their potential to proliferate and differentiate, which makes stem cell transplantation the method of choice to facilitate neural regeneration, modulate microenvironments, and replace injured tissues. Nearly four decades of experimental evidence have proven the efficacy and safety of stem cell therapies in pre-clinical animal tests and clinical trials (Borlongan, 2019). In this review, we discuss the potential mechanisms, cell types, methods, and time for stem cell transplantation, current trends in stem cell-based therapy, and the challenges that need to be overcome.

## Potential Mechanisms of Stem Cell Therapy for Ischemic Stroke

**Figure 1:**
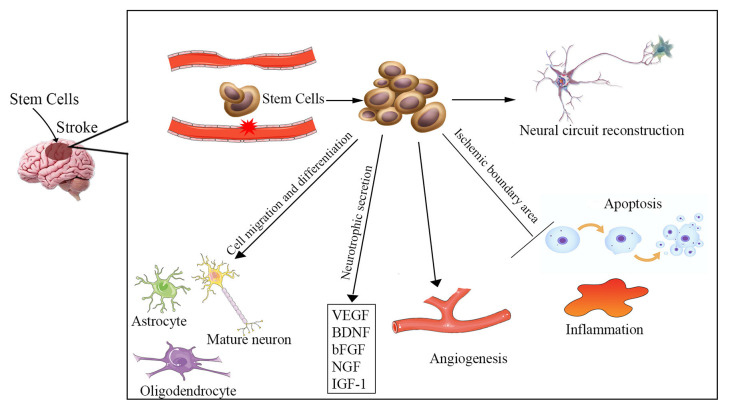
Overview of the potential mechanisms of stem cell therapy for ischemic stroke. Neuronal injury, increased apoptosis, inflammatory reaction, and vascular remodeling are involved in the pathophysiology of ischemic stroke. The underlying mechanisms of stem cell therapy for ischemic stroke may be to reverse these processes, including cell migration and differentiation into various cells to replace damaged neurons, neurotrophic secretion, apoptosis and inflammation inhibition, angiogenesis, and enhancement of neural circuit reconstruction. VEGF, vascular endothelial growth factor; BDNF, brain-derived neurotrophic factor; bFGF, basic fibroblast growth factor; NGF, nerve growth factor; IGF-1, insulin-like growth factor 1.

The etiology of ischemic stroke is due to a thrombotic or embolic blockage of an artery, resulting in acute loss of neurons, microglia, astrocytes, and oligodendroglia, as well as disruption of synapse structure. The pathophysiology of ischemic stroke remains unclear and involves a complex process. Increased apoptosis, inflammatory reaction, vascular remodeling, and neuronal injury are involved in ischemic stroke-induced neuronal death in the brain. Multiple potential mechanisms are involved in stem cell-based therapy for ischemic stroke (Figure 1), including cell migration and neurotrophic secretion, apoptosis and inflammation inhibition, angiogenesis, and neural circuit reconstruction. Therefore, stem cell therapy may be effective for stroke patients by replacing damaged neurons and promoting synaptic formation, as well as by stimulating angiogenesis, anti-apoptosis, and anti-inflammatory effects.

### Cell Migration and Neurotrophic Secretion

It has been proven that the adult brain is capable of self-repair *via* endogenous generation of new neurons to replace neurons that have died (Arvidsson et al., 2002). However, the survival rate and the total number of new neurons are extremely low. Moreover, there is insufficient neurogenesis to replace the lost neurons. Providing enough exogenous stem cells may be more conducive for repairing the injured neurons. The blood-brain barrier (BBB) is disrupted after a stroke. The transplanted stem cells can easily cross the BBB to gather in the infarcted brain areas and reconstruct the BBB integrity (Bang et al., 2017; Sun et al., 2020). Those cells can differentiate into various types of cells forming nervous tissue (e.g., mature neurons, oligodendrocytes, and astrocytes) and release a host of neurotrophic factors and cytokines [e.g., vascular endothelial growth factor (VEGF), brain-derived neurotrophic factor (BDNF), basic fibroblast growth factor (bFGF), nerve growth factor (NGF), insulin-like growth factor 1 (IGF-1)], which could promote neurogenesis to replace injured cells and improve neurological function (Ishibashi et al., 2004; Schinköthe et al., 2008; Kupcova Skalnikova, 2013).

### Apoptosis and Inflammation Inhibition

Several studies have suggested that a reduction in apoptosis in the ischemic boundary area occurs following cellular therapy that is associated with improved neurological recovery in experimental models (Stonesifer et al., 2017; Sun et al., 2020). It was reported that the neuroprotective effects of human bone marrow mesenchymal stem cells (hMSCs) against cerebral ischemia could be antagonized by the apoptosis-related Bcl-2 antibody (Zhang et al., 2019). When hMSCs were co-cultured with oxygen-glucose deprived (OGD)-injured neurons, they triggered a series of events, including a reduction in rates of apoptosis and necroptosis, downregulation of the necroptosis-related receptor-interacting protein kinase 1 and 3, and deactivation of caspase-3, an enzyme involved in apoptosis (Kong et al., 2017). It was proven that stem cells could efficiently promote neurological functional recovery *in vivo* by preventing neuronal apoptosis (Sun et al., 2020). Also, cerebral ischemia was found to promote the release of damage-associated molecular patterns (DAMPs) and matrix metalloproteinases (MMPs), which led to a series of inflammatory responses such as astrocyte and microglia activation, proinflammatory cytokine and chemokine production, and infiltration of leukocytes and neutrophils (Jayaraj et al., 2019; Stanzione et al., 2020). Stem cells can orchestrate other cells to exert anti-inflammatory effects by decreasing their secretion of inflammatory markers such as interleukin 6 (IL-6), granulocyte colony-stimulating factor (G-CSF), interleukin 1 (IL-1), and tumor necrosis factor (TNF-α; Redondo-Castro et al., 2017). Stem cells can also secrete a wide range of other factors including anti-inflammatory cytokines, such as IL-10, IL-12, and TGF-β (Boshuizen and Steinberg, 2018).

### Angiogenesis

Both *in vitro* and *in vivo* studies show that transplanted stem cells could promote neovascularization, stimulate angiogenesis and produce several angiogenic factors, which can be beneficial to functional recovery and neuronal regeneration (Zhang et al., 2011; Hicks et al., 2013; Zong et al., 2017). Transplanted stem cells can promote the secretion of VEGF and bFGF, which could enhance angiogenesis. Ryu et al. (2019) reported that *in vivo* treatment with MSCs significantly increased vessel length, vessel area, vessel volume, and the number of branching points. Kikuchi-Taura et al. (2020) demonstrated that gap junction-mediated cell-cell interaction was the prominent pathway for bone marrow mononuclear cells to activate angiogenesis after ischemia.

### Neural Circuit Reconstruction

Axonal plasticity may be the basic mechanism of stem cell therapy (Boshuizen and Steinberg, 2018). After stem cell treatment, the numbers of axons and myelin sheaths increase in the rat hippocampus, corpus callosum, and corpus striatum (Li et al., 2016). Andres et al. (2011) proved that human neural precursor cell transplantation could promote dendritic plasticity and axonal rewiring, which are mediated by the secretion of VEGF. Stem cells can interact with the surrounding neural tissues and can enhance graft-host synaptic connectivity to form new neural circuity (Oki et al., 2012).

## Stem Cell Types for Treatment of Ischemic Stroke

**Figure 2:**
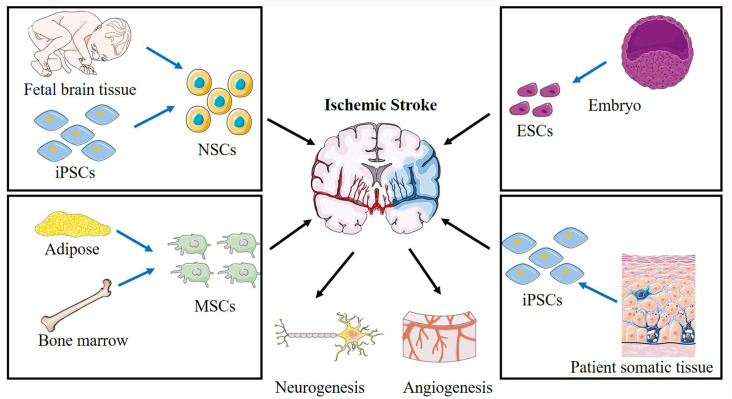
Schematic of various stem cells applied in cerebral ischemic stroke and the proposed mechanisms. Different sources of NSCs, ESCs, MSCs, BMMCs, iPSCs are applied to treat ischemic stroke. NSCs, neural stem cells; ESCs, embryonic stem cells; MSCs, mesenchymal stem cells; iPSCs, induced pluripotent stem cells.

Stem cells are characterized by two unique properties: the capacity for self-renewal and differentiation into other cell types. In animal models, several types of stem cells were reported as efficient treatments for stroke (Figure 2), including neural stem cells (NSCs), neural progenitor cells (NPCs), embryonic stem cells (ESCs; Hicks et al., 2009), mesenchymal stem cells (MSCs; Steinberg et al., 2016), bone marrow mononuclear cells (BMMCs; Prasad et al., 2014), and induced pluripotent stem cells (iPSCs; Tornero et al., 2013). In clinical trials, the most commonly used stem cells are MSCs or NSCs, including wild-type and genetically modified cells.

### Embryonic Stem Cells (ESCs)

ESCs are derived from the inner cell mass of blastocysts. They can differentiate into neurons, astrocytes, oligodendrocytes, and glial cells (Thomson et al., 1998; Wichterle et al., 2002). An advantage of using ESCs is their unlimited proliferative capacity *in vitro*. Several studies have demonstrated that the transplantation of differentiated ESCs can contribute to vascular regeneration and improved sensorimotor function after stroke (Oyamada et al., 2008; Hicks et al., 2009). However, ethical concerns, limited access to these cells, and the chances of forming tumors restrict widespread medical use of ESCs.

### Endogenous Neural Stem/Precursor Cells (NSCs)

NSCs are found in the embryonic developing brain and the adult mammalian brain. They are present within specific regions, including the ventricular-subventricular zone (V-SVZ) of the lateral ventricles (Alvarez-Buylla and Garcia-Verdugo, 2002), the subgranular zone (SGZ) of the hippocampus (Djavadian, 2004), and the external germinal layer (EGL) of the cerebellum (Hatten and Heintz, 1995). In recent years, numerous studies have shown that NSCs within the V-SVZ/SGZ/EGL undergo continuous proliferation and differentiation in the middle cerebral artery occlusion (MCAO) animal model for at least 4 months following stroke ischemia (Thored et al., 2006). Several methods are available for the isolation and culture of NSCs *in vitro*. NSCs can be isolated directly from the SVZ/SGZ/EGL in adults or from the neuroectoderm of the developing embryonic brain (Martí-Fàbregas et al., 2010; Guo et al., 2012). Although this method is the most convenient, the number and survival rate of neurons from these proliferative cells were extremely low. This is likely to be because of an increased concentration of inflammatory cytokines or inadequate nutritional support. Fortunately, various approaches can promote endogenous neurogenesis by promoting endogenous NSC survival, proliferation, and differentiation, which have provided a promising approach for treating stroke. For example, direct injection of certain growth factors, such as BDNF or VEGF, promotes the migration of endogenous NSCs to injured brain areas (Jin et al., 2002).

### Mesenchymal Stem Cells (MSCs)

Numerous pre-clinical and clinical studies of stroke have been carried out using MSCs (Zheng et al., 2018). MSCs can be easily isolated from bone marrow, adipose tissue, peripheral blood, umbilical cord, dental pulp, and amniotic fluid (Yan et al., 2013). Bone marrow-derived MSCs (BMSCs) are the most frequently used MSCs in experimental studies exploring stroke treatments. BMSCs can be autologous and thereby avoid immune rejection and viral transmission (Bang et al., 2005). BMSCs have been considered the gold standard for cell therapy research. Several studies have proven that BMSCs have the potential to differentiate into neuronal cells *in vitro* upon treatment with various growth inducers (Ferroni et al., 2013; Yue et al., 2016). After MSCs transplantation, the microenvironment of damaged brain tissues can be modulated toward a more regenerative and anti-inflammatory *milieu* by decreasing the release of pro-inflammatory cytokines, such as IL-1β, TNF-α, and IL-6, or by secretion of anti-inflammatory factors. Also, the secretion of trophic factors and antiapoptotic molecules that promote neurogenesis and angiogenesis is increased. Some studies have also reported the use of human umbilical cord-derived MSCs for ischemic stroke treatment (Feng et al., 2020; Noh et al., 2020).

### Bone Marrow Mononuclear Cells (BMMCs)

BMMCs are a heterogeneous population of cells that include monocytes, lymphocytes, hematopoietic stem cells, and mesenchymal, hematopoietic, and endothelial progenitor cells. BMMCs can be isolated autologously and conveniently cultured, which could be advantageous in acute ischemic stroke. In one clinical trial, it was reported that intravenous reinfusion of autologous BMMCs within 24–72 h of stroke onset might be feasible and effective (Savitz et al., 2011). Earlier transplantation of BMMCs may be more efficacious in enhancing recovery following stroke onset.

### Induced Pluripotent Stem Cells (iPSCs)

iPSC are a type of pluripotent stem cell that can be generated directly from somatic cells. iPSC have been shown to differentiate into neuronal and glial phenotypes, and could potentially be used for the treatment of stroke. However, the process of obtaining iPSCs is expensive and time-consuming. Furthermore, the translational use of iPSCs has been limited due to the potential risk of oncogenesis and insertional mutagenesis, poor integration into host neuronal circuits, and production of immune-tolerable cells (Kooreman and Wu, 2010).

## Stem Cell Transplantation

The most appropriate route for stem cell delivery remains unresolved. Cells can be transplanted using various delivery routes, including intracerebral, subarachnoid, and intranasal administration, as well as intravascular delivery *via* the tail vein (IV) or intra-artery (IA) injection. Among those methods, intracerebral administration is the most effective but most invasive method for exogenous stem cells to reach the injured region directly. Conversely, intravascular administration (IV or IA) is the least effective and least invasive method. Several concerns must be addressed in using the intracerebral or intraventricular method, including poor cell viability, invasiveness, immune rejection, and an uncertain prognosis, which present hurdles in the translational application of cell therapy (Wu et al., 2015). Regarding intravascular delivery methods, viable stem cells may not pass filtering organs, such as the lungs, liver, and spleen after IV administration. However, some studies promote the use of IA for stem cell administration as the shortest route toward the lesion, with improving cell engraftment and survival (Na Kim et al., 2017). The current animal studies or clinical trials did not systematically assess or report adverse events associated with the delivery route chosen. Only two animal studies explored the safety of the IA injection approach (Janowski et al., 2013; Yavagal et al., 2014). It has been reported that a higher incidence of strokes was observed due to microthrombus when injecting cells at a higher dose (2 × 10^6^) but the same was not observed when a lower dose (1 × 10^6^) was used to inject rats. However, this approach has not yet been tested in humans. Most of the current studies were conducted in animal models (Table 1; Brenneman et al., 2010; Chang et al., 2013; Kawabori et al., 2013; Cheng et al., 2015; Webb et al., 2018; Tian et al., 2019; Tobin et al., 2020; Asgari Taei et al., 2021). Further studies are needed to determine the best route for stem cell transplantation in treating stroke patients.

**Table 1:** Experimental study for various stem cells transplantation into the ischemic brain.

References	Cell type	Animal model	Route of administration	Delivery timing (after ischemia)	Dose	Results
Asgari Taei et al. (2021)	Human ESC	Rat MCAO	left lateral ventricle	1, 24, and 48 h	1 × 10^6^	Neurological deficits↓ Synaptic↑ Infarct volumes↓ Cerebral edema↓
Cheng et al. (2015)	NSC line C17.2	Rat MCAO	IV (tail vein)	24 h	5 × 10^6^	Functional recovery↑ Neurogenesis↑ proliferation↑
Tian et al. (2019)	Leukemia inhibitory factor -transfected NSCs	Rat MCAO	IV (tail vein)	2 h	5 × 10^5^	Lesion volume↓ Functional↑ Glial cell regeneration↑ Apoptosis↓
Webb et al. (2018)	NSCs-derived extracellular vesicle	porcine MCAO	IV	2, 14, and 24 h	2.7 × 10^6^	Lesion volume↓ Cerebral diffusivity↑ Motor activity ↑ Exploratory behavior↑
Tobin et al. (2020)	interferon-γ–activated BMSCs	Rat MCAO	IV	4.5 h	5 × 10^6^/kg	Functional recovery↑ Oligodendrogenesis↑ Microglia activation↓ Inflammation↓
Kawabori et al. (2013)	Rat BMSCs	Rat MCAO	ipsilateral striatum	1 or 4 weeks	1 × 10^5^ or 1 × 10^6^	Functional recovery↑ Differentiation ↑
Brenneman et al. (2010)	Autologous BMMCs	left MCA and left ICA occlusion	IA (carotid artery)	24 h	1 × 10^7^ or 4 × 10^6^	Neurological recovery↑ Infarct volume↓ Inflammation↓
Chang et al. (2013)	Human iPSCs	Rat MCAO	intracerebral	7 days	2 × 10^5^	Behavioral↑ Neurogenesis↑ Inflammation↓ apoptosis↓

*ESCs, embryonic stem cells; NSCs, neural stem cells; BMSCs, bone marrow-derived mesenchymal stem cells; BMMCs, bone marrow mononuclear cells; iPSCs, induced pluripotent stem cells; MCAO: middle cerebral artery occlusion*.

The appropriate timing of transplantation after ischemia is another critical factor affecting treatment outcomes and the survival of transplanted cells. In clinical trials, the time window of stem cell transplant administration ranged from 24 h to 2 years. Depending on the cell type or source, different time windows of administration possibly contribute to various levels of efficacy (Li et al., 2020). Several studies using the MCAO animal model have reported stem cell transplantations at times of 1 day (Chu et al., 2004; Zhang et al., 2009), 7 days (Kelly et al., 2004; Daadi et al., 2008), or 4 weeks (Jin et al., 2010) after the stroke. However, no studies have compared the effects of injection at different times. There are diverse opinions and a lack of consensus on the optimal timing of transplantation after stroke onset.

## Current Trends in Stem Cell Therapy

**Figure 3:**
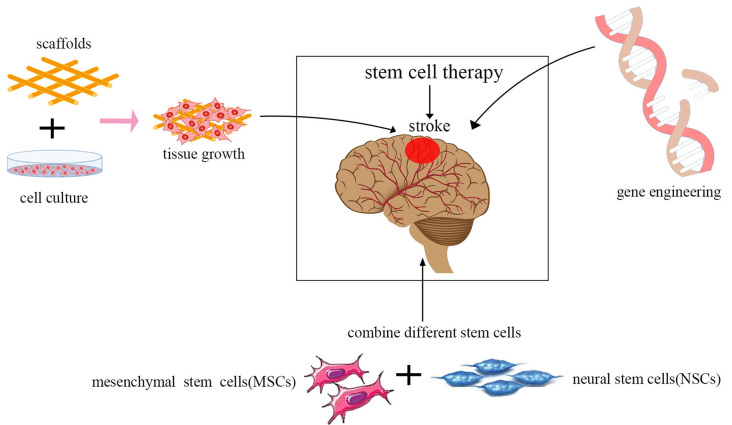
Current trends in stem cell therapy for ischemic stroke. The integration of gene therapy, tissue engineering scaffolds, and the use of various stem cell types are the current trends in stem cell therapy.

In recent years, stem cell therapeutics have become increasingly effective for ischemic stroke with the advancement of technology including the use of genetic and tissue engineering. There are some combination therapy strategies, such as the integration of gene therapy, tissue engineering scaffolds, and the use of various stem cell types (Figure 3).

Gene therapy is designed to introduce therapeutic genes into target cells. Transplantation of stem cells whose genes have been modified using viruses to express neurotrophic and growth factors, such as BDNF, VEGF, NGF, GDNF, Noggin, placental growth factor (PIGF), hepatocyte growth factor (HGF), erythropoietin (EPO), angiopoietin-1 (ANG-1), and IL-10 has been proven to be more effective in promoting neuronal tissue regeneration compared to the unmodified stem cells in experimental stroke animal models (Wang et al., 2004; Ding et al., 2011; Chen et al., 2013; Nakajima et al., 2017; Wang et al., 2018; Moxon et al., 2019). Bernstock et al. (2019) showed that NSCs modified by the small ubiquitin-like modifier (SUMO) could increase cell survival and enhance neuronal differentiation. Viral vectors are promising tools for the genetic modification of cells by integrating into the genome. However, the risk of oncogenesis can be increased greatly by altering the host gene expression patterns. Thus, more experimental animal studies are needed to evaluate systematically the safety of genetically modified stem cell therapy before this method is approved for wide use in clinical studies.

Stem cells combined with tissue engineering approaches are becoming increasingly popular in regenerative medicine, as well as for repairing the damaged central nervous system. Biological scaffolds can be modified and designed to meet most of the requisites of various tissues. They could promote the survival of cells. They could promote astrocyte infiltration into the stroke cavity rather than glial scar formation post-injury (Nih et al., 2017). An ideal tissue-engineered scaffold should have adequate histocompatibility and have a three-dimensional architecture that provides an ideal microenvironment for supporting cell adhesion, migration, proliferation, and differentiation without eliciting inflammatory responses *in vivo*. Furthermore, many biomaterials can reversibly bind to growth factors. Bible et al. (2009) reported that plasma polymerized allylamine (ppAAm)-treated poly-D, L-lactic-co-glycolic acid (PLGA) scaffold particles could attach NSCs *in vitro* at high density, and act as structural support for NSCs injected directly into the lesion cavity *in vivo*. Zhang et al. (2017) showed that transplantation of BMSCs in combination with a plasma scaffold into the cystic cavity after focal cerebral ischemia significantly reduced the infarct legion region, and motor function was dramatically improved. Moshayedi et al. (2016) reported that HA hydrogels could enhance the survival of transplanted cells and promote astrocytic differentiation *in vivo*.

Using a combination of multiple types of stem cells is another trend in cell therapy. Several studies have shown that various types of stem/progenitor cells and their derivatives can be more effective than a single type of stem cell for the reconstruction of damaged neural tissue after ischemic stroke, as different neurotrophic factors could be secreted by different cells. However, the detailed mechanism of this method remains unexplored, as well as the optimal composition of cell types, dose, and density to achieve the best neurofunctional recovery for ischemic stroke. In the study by Hosseini et al. (2015), MSCs from adult rat bone marrow were injected 1 day after middle cerebral artery occlusion (MCAO), and the NSCs from the ganglion eminence of a rat embryo at 14 days were transplanted 7 days after MCAO. The results showed that using a combination of MSCs and NSCs had a better neurological outcome, and fewer brain lesions were observed.

## Challenges to Overcome in The Clinical Application of Stem Cell Therapy

Before stem cells can be successfully used in clinical translation, many setbacks must be overcome. First, tracking of transplanted cells and poor survival. Cells can be tracked *in vivo* with magnetic resonance imaging (MRI), single-photon emission computed tomography (SPECT/CT), positron emission tomography (PET), and bioluminescence imaging (BLI) using a dual green fluorescent protein-Luciferase (GFP-Luc) reporter system (Manley and Steinberg, 2012; Zheng et al., 2017). The work of Moshayedi et al. (2016) demonstrated that hyaluronic acid (HA) hydrogels can enhance the survival of transplanted cells for at least 6 weeks and can be tracked *in vivo* by MRI. Second, the clinical application of stem cells raises numerous ethical concerns, particularly for NSCs and ESCs. The application of iPSCs may avoid this problem. Additionally, the production of autologous iPSCs is feasible, but it is rather costly and would take several months for its production before the cells could be used for transplantation. Moreover, the clinical application of stem cells may raise numerous safety concerns. Allografts may cause immune rejection. Stem cells have the potential to differentiate into undesired tissues and can promote tumor growth and metastasis by enhancing the generation of new blood vessels and altering the tumor microenvironment (Lazennec and Jorgensen, 2008; Patel et al., 2010). Erdö et al. (2003) demonstrated that contamination of undifferentiated ES cells could promote tumorigenesis. Amariglio et al. (2009) reported that a patient with ataxia-telangiectasia who was treated with intracerebellar and intrathecal injection of donor-derived neural stem cell transplantation developed a brain tumor 4 years after the first treatment. More clinical research is warranted to explore the optimal transplantation plan (i.e., timing, route, and dose of administration).

## Conclusion

Stem cells are very promising candidates for augmenting brain repair, to restore function following stroke treatment. During the last few years, various approaches to transplantation have been applied in ischemic stroke animal models and clinical trials. The outcomes of these studies have been encouraging, with the transplanted stem cells having various beneficial effects, including the reduced neurological deficit, reduced infarct area, reduced inflammation, and increased neurogenesis and angiogenesis. However, the clinical application of stem cell-based therapies remains in its infancy. Many issues still need to be resolved, including efficacy, safety, and feasibility. With the continuous exploration of regenerative medicine and the development of cell transplantation techniques, stem cell therapy will help ischemic stroke patients achieve better neurofunctional recovery soon.

## Author Contributions

HL and YH conceived the main outline. HL wrote the manuscript. XZ and HC made the table and figure. SR, CL and YO took charge of manuscript revision in English. YH participated in the correction and final inspection of this review. All authors contributed to the article and approved the submitted version.

## Conflict of Interest

The authors declare that the research was conducted in the absence of any commercial or financial relationships that could be construed as a potential conflict of interest.
